# Breaking host barriers: detection of *Cytauxzoon banethi* in a non-felid feliform, the striped hyena (*Hyaena hyaena*)

**DOI:** 10.1016/j.ijppaw.2026.101268

**Published:** 2026-07-29

**Authors:** Alireza Sazmand, Angela Monica Ionică, Leili Moradi, Mohammad Babaei, Georgiana Deak, Domenico Otranto, Andrei Daniel Mihalca

**Affiliations:** a Department of Pathobiology, Faculty of Veterinary Medicine, Bu-Ali Sina University, Hamedan, 6517658978, Iran; b Department of Parasitology and Parasitic Diseases, University of Agricultural Sciences and Veterinary Medicine of Cluj-Napoca, Calea Mănăştur 3-5, Cluj-Napoca, 400372, Romania; c Clinical Hospital of Infectious Diseases of Cluj-Napoca, 23 Iuliu Moldovan, Cluj-Napoca, 400348, Romania; d Department of Basic Sciences, Faculty of Veterinary Medicine, Bu-Ali Sina University, Hamedan, 6517658978, Iran; e Department of Veterinary Medicine, University of Bari, Bari, 70010, Italy; f Department of Veterinary Clinical Sciences, City University of Hong Kong, Kowloon Tong, Hong Kong, China; g Chulalongkorn University, Bangkok, Thailand

**Keywords:** Cytauxzoonosis, Striped hyena, *Hyaena hyaena*, Piroplasm, Wildlife parasite, Iran, Host expansion, Phylogeny

## Abstract

*Cytauxzoon banethi* is one of the least characterized members of the genus *Cytauxzoon* and has so far been reported only from two European wildcats (*Felis silvestris*) in Romania. Consequently, its host range and geographic distribution remain poorly understood. In the present study, we molecularly characterized a *Cytauxzoon* isolate detected in a free-ranging striped hyena (*Hyaena hyaena*) from western Iran. Genomic DNA extracted from blood was analyzed by PCR amplification and sequencing of partial 18S rRNA and mitochondrial cytochrome b (*cytb*) genes. The 18S rRNA sequence was identical to published *C. manul* sequences and highly similar to several other *Cytauxzoon* species, confirming the limited species-level resolution of this conserved marker. In contrast, direct comparison of the *cytb* sequence with the available *C. banethi* reference sequence revealed only five synonymous nucleotide substitutions across 1,089 aligned positions (99.5% identity). Phylogenetic analysis of the *cytb* dataset placed the obtained isolate within the *C. banethi* lineage as the sister taxon to *C. manul*, supporting its identification as *C. banethi*. To the best of our knowledge, this represents the first molecular detection of *C. banethi* in a member of the family Hyaenidae, the first record in a non-felid feliform host, the first report from Iran and Asia, and the first detection of this species outside Europe. These findings expand the currently recognized host range and geographic distribution of *C. banethi* and emphasize the importance of multilocus molecular approaches and broader wildlife surveillance for improving our understanding of the diversity and evolution of *Cytauxzoon* species in general, and for better assessing the genetic diversity and taxonomic boundaries of *C. banethi*.

## Highlights

• Molecular characterization of a *Cytauxzoon* sp. isolated from a free-ranging striped hyena (*Hyaena hyaena*) in western Iran
• First molecular detection of *C. banethi* in a member of the family Hyaenidae
• First report in a non-felid feliform host
• First record from Iran and Asia
• First detection of this parasite outside Europe

## Graphical abstract

**Figure:**
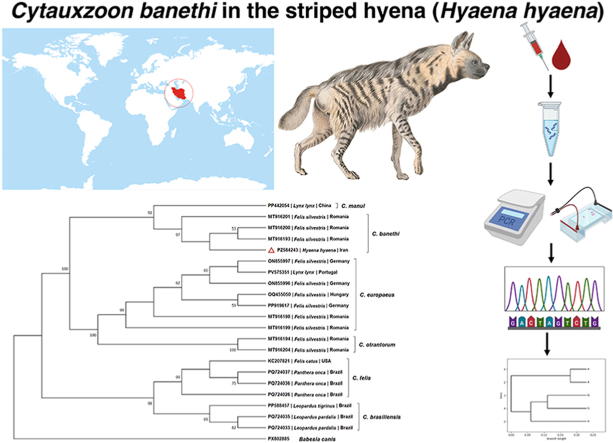

## 1 Introduction

Piroplasmids of the genus *Cytauxzoon* are tick-transmitted apicomplexan parasites that infect wild and domestic felids worldwide (Schnittger et al., 2022). While *Cytauxzoon felis* is recognized as an important pathogen of domestic cats in North America, an increasing number of molecular studies have revealed a growing diversity of *Cytauxzoon* species circulating among wild felids across Europe, Asia and South America (Birkenheuer et al., 2006; Chong et al., 2025; Cui et al., 2024; Duarte et al., 2024; Meinkoth and Kocan, 2005; Panait et al., 2021; Reichard et al., 2005; Willi et al., 2022). In particular, three novel species, *i.e*., *Cytauxzoon europaeus*, *Cytauxzoon otrantorum*, and *Cytauxzoon banethi*, have been described in European wild felids based on molecular and phylogenetic evidence (Panait et al., 2021). To date, *C. banethi* has been reported only from two European wildcats (*Felis silvestris*) in Romania (Panait et al., 2021). As all known records originate from a single felid host species, it remains unclear whether *C. banethi* is restricted to members of the family Felidae or circulates more broadly among other feliform carnivores.

The striped hyena (*Hyaena hyaena*) is a widespread carnivore distributed across parts of Africa and Asia (Wilkinson et al., 2024). Despite its ecological importance and taxonomic placement within the Feliformia, its parasitic fauna has received relatively little attention, with no molecularly confirmed record of *Cytauxzoon* spp. reported from this host. The present study aimed to molecularly characterize a *Cytauxzoon* isolate detected in a free-ranging striped hyena from Iran using genetic mitochondrial (i.e., *cytb*) and ribosomal (i.e., *18S rRNA*) markers and to determine its phylogenetic relationships with other members of the genus *Cytauxzoon*.

## 2 Methods

### 2.1 Sample collection

The carcass of an adult female striped hyena (*Hyaena hyaena*) from Bahar County, Hamedan Province, western Iran, was found by one of the co-authors and transferred to the Faculty of Veterinary Medicine, Bu-Ali Sina University in July 2023. According to local reports, the animal had been shot and then attacked by local residents following alleged predation on livestock. During the post-mortem examination, whole blood was collected from the heart and preserved for molecular investigation of haemoprotozoan parasites. It is worth noting that the striped hyena is a protected wildlife species in Iran and is the only extant member of the family Hyaenidae in the country. However, under local laws, ethical approval was not required for the presentation of the carcass for necroscopsy examination.

### 2.2 DNA isolation, molecular analysis, and sequencing

Genomic DNA was extracted from 200 μL EDTA-treated blood samples using the MBST DNA Kit (MBST, Tehran, Iran) according to the manufacturer's instructions. Initially, the samples were tested for piroplasms by conventional polymerase chain reaction (cPCR) using primers GF2: GTCTTGTAATTGGAATGATGG and GR2: CCAAAGACTTTGATTTCTCTC, targeting a 560–610 bp fragment of 18S rRNA (Bonnet et al., 2007). The cPCR product was visualized on a UV imager (Transilluminator, Vilber Lourmat, France) after electrophoresis in a 1% agarose gel (SinaClon, Tehran, Iran) stained with DNA Safe Stain (SinaClon, Tehran, Iran). Sequencing was performed on an Applied Biosystems 3500 Genetic Analyzer (Thermo Fisher Scientific, MA, USA) at the Codon Genetics Laboratory (Tehran, Iran). The sequence was compared with other sequences in the NCBI GenBank® database using the Basic Local Alignment Search Tool (BLAST). Upon identification of *Cytauxzoon* DNA, the specimen was further examined for longer fragments of *cytb* and *18S rRNA* by nested PCR, using primers and protocols described by Panait et al. (2021) (Table 1).

**Table 1:** Primers used during the current study.

Target gene	Primer (nucleotide sequence 5’ – 3′)	Product (bp)	Reference
*18S rDNA*	Nest 1 7549F: GTCAGGATCCTGGGTTGATCCTGCCAG	1726	Millán et al. (2007)
	7548R: GACTGAATTCGACTTCTCCTTCCTTTAAG		
	Nest 2 Cyt-SSU-F2: CATGGATAACCGTGCTAATTG	1335	Panait et al. (2021)
	Cyt-SSU-R4: AGGATGAACTCGATGAATGCA		
*Cytochrome b* (*cytb*)	Nest 1 Cytaux_cytb_F1: CTTAACCCAACTCACGTACC	1434	Schreeg et al. (2013)
	Cytaux_cytb_R3: GGTTAATCTTTCCTATTCCTTACG		
	Nest 2 Cytaux_cytb_Finn: ACCTACTAAACCTTATTCAAGCRTT	1333	Panait et al. (2021)
	Cytaux_cytb_Rinn: AGACTCTTAGATGYAAACTTCCC		

The bands obtained were excised from the gel, purified using a commercial kit (Gel/PCR DNA Fragment Extraction Kit, Geneaid Biotech, Taiwan), and bidirectionally sequenced. The obtained sequences were assembled and edited using Geneious software (Biomatters Ltd., New Zealand) and compared with other GenBank® entries using BLAST. For similarity analysis, manual alignments were also performed against *C. banethi* and *C. otrantorum* sequences, for which the *cytb* sequences are listed as “UNVERIFIED”, thus being disregarded by the standard BLAST algorithm.

### 2.3 Phylogenetic analyses

The phylogenetic analysis was performed using MEGA X software (Kumar et al., 2018). The sequences were aligned with other GenBank® entries by the MUSCLE algorithm, and the alignments were trimmed at both ends in order to ensure the same length for all included sequences. Thus, the final datasets included 1,057 positions. The best model was selected by the software based on the lowest Bayesian Information Criterion (BIC) score. A Maximum Likelihood method was used, with the following parameters: Hasegawa-Kishino-Yano model (Hasegawa et al., 1985), with a discrete Gamma distribution used to model evolutionary rate differences among sites (5 categories (+*G*, parameter = 0.6310)).

## 3 Results

PCR amplification successfully generated partial *cytb* and 18S rRNA sequences from a *Cytauxzoon* parasite in the striped hyena sample. Standard BLAST searches of the *cytb* sequence showed the highest similarity to *C. manul* (89.47% with PX924971 and 88.81% with PP442054), followed by *C. europaeus* (81.00–81.62% with several isolates, e.g., ON856037 and PP910609). However, the available *C. banethi cytb* reference sequence (MT916193) is listed as an unverified GenBank entry and therefore is not retrieved by standard BLAST searches. Consequently, a direct pairwise sequence alignment was performed against this reference sequence, revealing only five synonymous nucleotide substitutions across 1,089 aligned positions (99.5% sequence identity). Translation of the nucleotide sequence produced an uninterrupted open reading frame without internal stop codons, supporting the authenticity of the obtained sequence.

BLAST analysis of the long 18S rRNA sequence (1218 bp) showed complete identity with two published *Cytauxzoon* sequences assigned to *C. manul* (AY485690 and PZ387854) and 99.75% identity with another *C. manul* isolate (PZ387856). The sequence also showed 99.75% identity with other *Cytauxzoon* species, including *C. europaeus* (ON380447) and *C. otrantorum* (MT904033), differing by only three nucleotide substitutions. These findings are consistent with the highly conserved nature of the 18S rRNA gene and its limited resolution for distinguishing closely related *Cytauxzoon* species. The short 18S rRNA sequence (522 bp) was identical to the corresponding region of the long sequence (positions 342–863), including one of the three variable nucleotide positions.

**Fig. 1:**
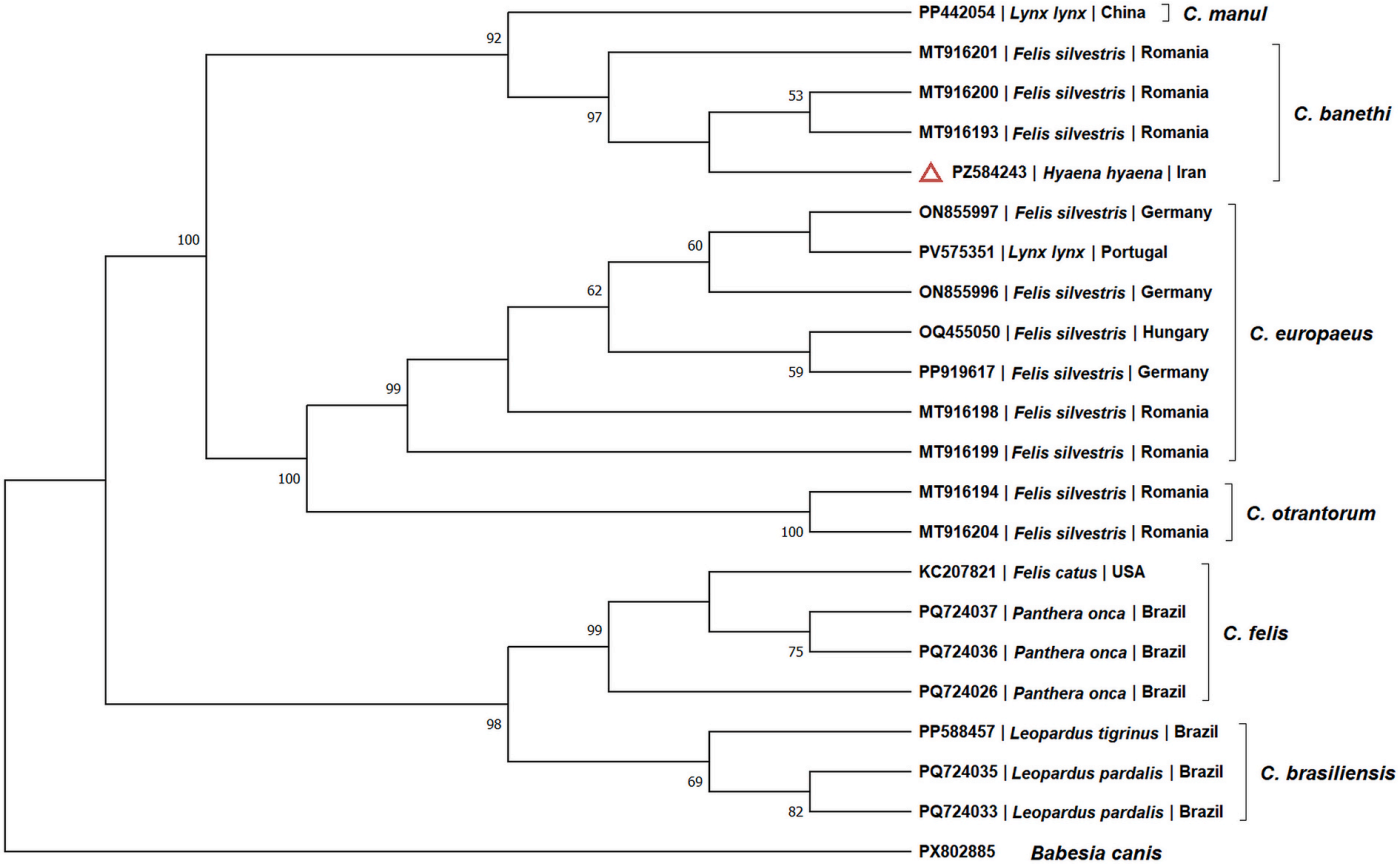
Bootstrap consensus tree of *cytb*, inferred from 1000 replicates. The percentage of trees in which the associated taxa clustered together is shown next to the branches (values below 50 not shown). This analysis involved 21 *Cytauxzoon* spp. nucleotide sequences and a *Babesia canis* sequence used as an outgroup. There were 1,057 positions in the final dataset.

**Fig. 2:**
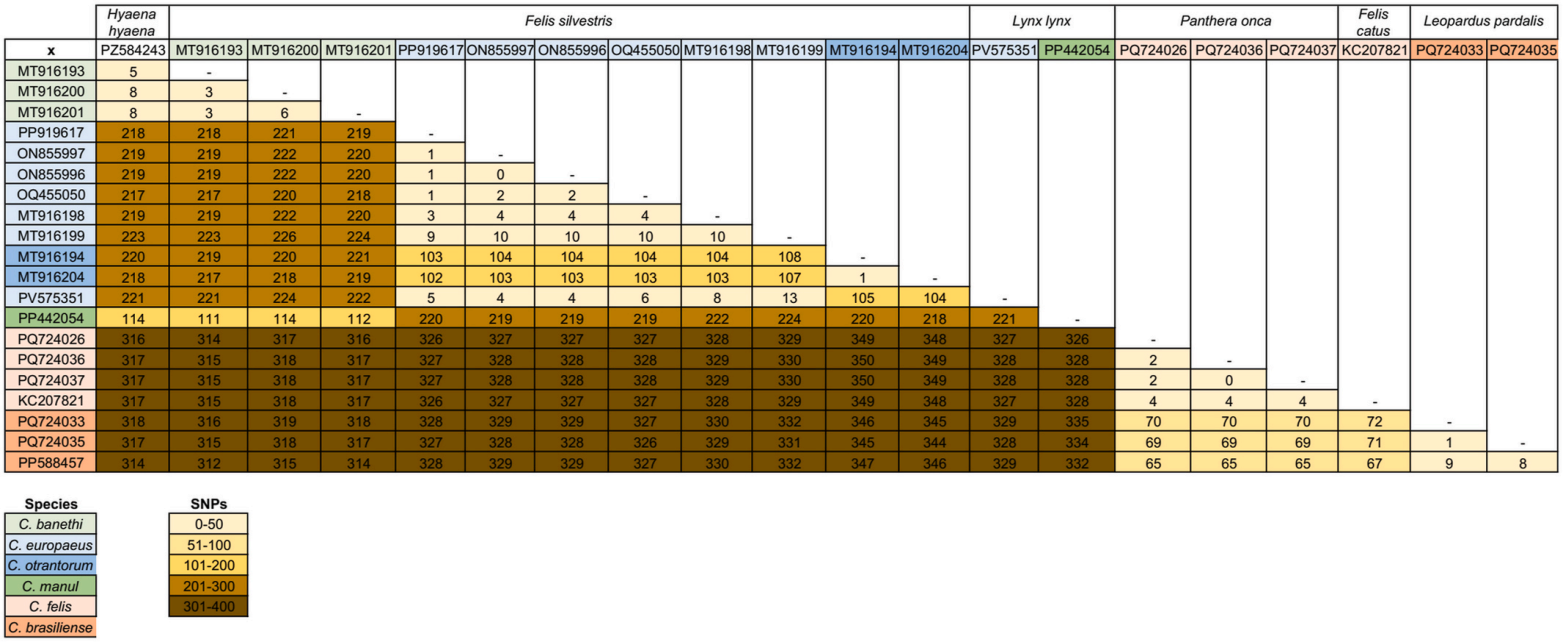
Distance matrix of the *Cytauxzoon* spp. sequences used for phylogenetic analysis of the partial *cytb* gene, generated using MEGA X software. Column and rows' names are represented by the GenBank Accession Number. The numbers within the table represent the actual number of inter-sequence single nucleotide polymorphisms (SNPs). The host species are listed in the top row.

Phylogenetic analyses based on the *cytb* dataset placed the obtained sequence within the *C. banethi* lineage (Fig. 1), as the sister taxon to *C. manul*. Furthermore, intraspecific variation within the *cytb* dataset ranged from 6 to 13 nucleotide differences in *C. banethi* and *C. europaeus,* respectively (Fig. 2), further supporting the current specimen's identification as *C. banethi*. In contrast, phylogenetic analysis of the 18S rRNA gene was not performed due to its limited phylogenetic resolution, with interspecific variation ranging from 0 to a maximum of 4 nucleotide substitutions across all analyzed *Cytauxzoon* species except *C. felis*.

## 4 Discussion

The detection of *C. banethi* in a striped hyena represents not only an expansion of the host range but also the first molecular evidence of protozoon in the genus *Cytauxzoon* infection in a member of the family Hyaenidae. The parasite was consistently identified using multiple genetic loci, and phylogenetic analyses placed the sequences within the *C. banethi* clade. To the best of our knowledge, this is the first molecular detection of *C. banethi* in a member of the family Hyaenidae, the first report in a non-felid feliform host, and the first record of this species outside Europe.

*Cytauxzoon banethi* was originally described from two European wildcats (*Felis silvestris*) in Romania (Panait et al., 2021), and no additional hosts have been reported since. Consequently, knowledge of the parasite's biology, host specificity, and geographic distribution has remained extremely limited. The present finding substantially expands the known host range of *C. banethi* and suggests that its circulation may not be restricted to members of the family Felidae. Analogously, within piroplasmids, *Hepatozoon* and *Babesia* species have been reported from both felids and brown (*Parahyaena brunnea*) and spotted hyaenas (*Crocuta crocuta*) (Burroughs et al., 2017; East et al., 2008; Williams et al., 2014), suggesting ecological overlap, vector sharing, or historical host-switching events. Since infections with *Cytauxzoon* spp. have been almost exclusively associated with members of the family Felidae, our result may indicate a broader host range of this hemoparasite than previously recognized. This concept is of paramount importance for understanding the evolution and emergence of potential novel parasites undergoing host shifts from wildlife to domestic dogs and cats, driven by a complex interplay of biotic and abiotic factors (Rojas et al., 2026). Nonetheless, the evolutionary and ecological implications of this finding remain unclear. The striped hyena is an opportunistic scavenger with extensive movement patterns and frequent exposure to tick-infested environments, factors that may facilitate contact with a wide range of domestic and wildlife hosts and the vector-borne pathogens they carry. Whether striped hyenas are incidental hosts or participate in the natural maintenance cycle of *C. banethi* cannot be determined from a single case. Nevertheless, the molecular confirmation of infection indicates that the host range of this parasite warrants re-evaluation through targeted surveys of domestic and wild carnivores across different feliform lineages. Notably, in a previous study in Iran (Sazmand et al., 2025), none of the 774 blood DNA samples collected from cats across six Iranian provinces tested positive for piroplasms using two sets of primers targeting the *18S rDNA* gene (Bonnet et al., 2007; Tabar et al., 2008). Furthermore, sequencing of 430 bp amplicons produced by ITS-2-based primers (Brown et al., 2008) in nine samples failed and the samples were therefore considered negative. In contrast, two studies on domestic cats (Ebrahimi Toodarvari et al., 2025; Rahmati Moghaddam et al., 2020) and one study on a wildcat (*Felis silvestris*) (Zaeemi et al., 2015) reported the presence of *Cytauxzoon* sp. DNA in the blood of animals (Brown et al., 2008). As Sanger sequencing was not performed to confirm identification, and given that, based on current knowledge, *C. felis* is present only in the Americas (Alves et al., 2025; Duarte et al., 2024; Wikander and Reif, 2023), the detected pathogens could be *C. manul*, *C. banethi*, *C. europaeus*, or *C. otrantorum*, which have been reported from Asia and Europe.

The present study also substantially expands the known geographic distribution of *C. banethi*, which had previously been reported only in Romania. This apparent geographic restriction may therefore reflect limited sampling rather than a genuinely narrow distribution, as observed for *C. europaeus*, whose ranges have expanded substantially following increased molecular surveillance (Muñoz-Hernández et al., 2025). Future investigations with larger sample sizes, additional wildlife hosts, and potential tick vectors are required to clarify the epidemiology, host associations, and evolutionary history of *Cytauxzoon* spp. across its distribution.

This study also highlights the varying taxonomic resolution provided by the molecular markers used. The partial 18S rRNA sequence was identical to published *C. manul* sequences and highly similar to other *Cytauxzoon* species, confirming that this locus is highly conserved and provides limited resolution for distinguishing closely related taxa. In contrast, the mitochondrial cytb gene exhibited substantially greater discriminatory power, showing approximately 99.5% sequence identity with *C. banethi* and consistently placing the Iranian isolate within the *C. banethi* lineage in phylogenetic analyses. Therefore, species assignment in the present study relied primarily on the *cytb* marker rather than on the 18S rRNA gene alone.

Although the currently available molecular evidence strongly supports the identification of the parasite as *C. banethi*, additional mitochondrial or nuclear loci would further strengthen species-level assignment. The scarcity of reference sequences for *C. banethi*, particularly protein-coding genes, currently limits comprehensive multilocus comparisons. Future studies that include additional genetic markers and isolates from different hosts and geographic regions will improve our understanding of the genetic diversity and phylogenetic relationships of this species.

## 5 Conclusions

The molecular detection of *C. banethi* in a striped hyena from Iran provides the first evidence of infection in a non-felid feliform host and substantially expands the known geographic range of this parasite. These findings underscore the need to expand molecular surveillance of piroplasmids in understudied wildlife species and suggest that the diversity, host range, and distribution of *C. banethi* are currently underestimated.

## Consent to participate

Not applicable.

## Ethics approval

Under local laws, ethical approval was not required for necroscopic examination of a dead animal.

## Data availability

The datasets generated and analyzed in the current study are available on the NCBI GenBank Nucleotide platform (https://www.ncbi.nlm.nih.gov/genbank/) and can be accessed via accession numbers PZ556421 and PZ584243. Any additional data is available from the corresponding author on request.

## Declaration of generative AI and AI-assisted technologies in the writing process

During the preparation of this work, ChatGPT and Grammarly were used to improve readability and to correct grammatical errors, punctuation, and typing mistakes. After using these tools, the authors reviewed and edited all content as needed and take full responsibility for the publication's content.

## Funding

This work was supported by a grant from 10.13039/501100009694Bu-Ali Sina University (Grant Number: 1402–1490). During this study, Georgiana Deak was supported by a grant from the 10.13039/100018987Ministry of Research, Innovation and Digitization, CNCS-UEFISCDI, project number PN-IV-P2-2.1-TE-2023-0054, within PNCDI IV.

## CRediT authorship contribution statement

**Alireza Sazmand:** Conceptualization, Funding acquisition, Investigation, Project administration, Resources, Supervision, Writing – original draft, Writing – review & editing. **Angela Monica Ionică:** Investigation, Writing – original draft, Writing – review & editing. **Leili Moradi:** Investigation. **Mohammad Babaei:** Investigation. **Georgiana Deak:** Writing – review & editing. **Domenico Otranto:** Writing – review & editing. **Andrei Daniel Mihalca:** Funding acquisition, Supervision, Writing – review & editing.

## Declaration of competing interest

The authors declare that they have no known competing financial interests or personal relationships that could have appeared to influence the work reported in this paper.
